# Urinary Function of the Sasang Type and Cold-Heat Subgroup Using the Sasang Urination Inventory in Korean Hospital Patients

**DOI:** 10.1155/2020/7313581

**Published:** 2020-09-09

**Authors:** Seul Lee, Yongjae Lee, Sang Yun Han, Nayoung Bae, Minwoo Hwang, Jeongyun Lee, Han Chae

**Affiliations:** ^1^School of Korean Medicine, Pusan National University, Yangsan 50612, Republic of Korea; ^2^Department of Sasang Constitutional Medicine, Pusan National University Korean Medicine Hospital, Yangsan 50612, Republic of Korea; ^3^Department of Sasang Constitutional Medicine, Division of Clinical Medicine, School of Korean Medicine, Pusan National University, Yangsan 50612, Republic of Korea; ^4^Department of Medical Education, Seoul National University College of Medicine, Seoul 03080, Republic of Korea; ^5^Department of Sasang Constitutional Medicine, College of Korean Medicine, Kyung Hee University, Seoul 02447, Republic of Korea

## Abstract

**Introduction:**

The Sasang type-specific pathophysiological symptom is pivotal for the Sasang type classification and pattern identification. The Sasang Urination and Defecation Inventory (SUDI) for urinary function analysis was developed; however, the clinical usefulness of urination-related subscales of SUDI in the Sasang type and Cold-Heat subgroup was not reported with acceptable validation analysis.

**Methods:**

The clinical diagnosis of the Sasang type and Cold-Heat subgroup, responses to SUDI items, and weight and height of the 350 hospital patients were acquired retrospectively. The Sasang Urination Inventory (SUI) with SUI-CHR (problematic physical characteristics of urine), SUI-HSS (hypersensitivity of urinary urgency and high frequency), and SUI-DIS (urinary discomfort of hesitancy and residual urine sense) subscales using 12 items of SUDI were improvised. The item and construct validity of the SUI were examined using item response theory and confirmatory factor analysis, and the clinical usefulness of the SUI in Sasang type and Cold-Heat subgroup differentiation was attested.

**Results:**

The SUI and its subscales showed acceptable structural validity and have clinical usefulness in the Tae-Eum type. The Tae-Eum type has a significantly higher SUI-CHR score than did the So-Yang type, and the Heat subgroup has a significantly higher SUI-HSS score than did the Cold subgroup in the Tae-Eum type. *Discussion*. The distinctive Sasang type- and Cold-Heat subscale-specific pathological symptoms in urinary function were revealed using the SUI. The SUI combined with objective Sasang typology measures might be useful for integrative precision medicine combining Eastern and Western practice and for evidence-based clinical education for medical professions.

## 1. Introduction

The Sasang typology divides persons into four groups of Tae-Yang, So-Yang, Tae-Eum, and So-Eum Sasang types based on their typical psychological, physical, and pathological features [1, 2] and subsequently suggests safe and effective type- and subgroup-specific treatment using acupuncture [3] and medical herbs [4].

Previous studies on the Sasang typology showed the biopsychological characteristics [5, 6] that the So-Yang type has high Sasang Personality Questionnaire (SPQ) [7, 8] total score, high NEO-Personality Inventory Extraversion score, high Temperament and Character Inventory (TCI) Novelty-Seeking, and low TCI Harm-Avoidance score, while the So-Eum type is on the contrary.

As for the physical features [9–11], the Tae-Eum type has high body mass index (BMI), high Ponderal Index (PI), high Sasang Digestive Function Inventory (SDFI) score, high parasympathetic reactivity, and low sympathetic reactivity, while the So-Eum type has opposite features.

Furthermore, there have been studies stating that specific disease is frequent in certain Sasang types. For instance, the Tae-Eum type has abdominal obesity [12], metabolic syndrome [13], hypertension [14], high insulin resistance, [15], and obstructive sleep apnea [16]. Moreover, the So-Eum type has psychological problems of depression [5] and post-traumatic stress disorder [17].

The Sasang type-specific pathophysiological symptoms are pivotal for Sasang type identification, clinical diagnosis of the Cold-Heat subtype, and disease remission and exacerbation [11]. Furthermore, five functional systems of digestion [9], urination and defecation [18], and sleep [16, 19] and perspiration [11] were suggested to be major categories of type-specific pathophysiological symptoms with high clinical practicality.

The SDFI [10] measuring the digestive function of the Sasang typology was developed based on systematic review [9], and it showed significant negative correlation with the Nepean Dyspepsia Index-Korean and Functional Dyspepsia-Related Quality of Life-Eating status and positive correlation with the Dutch Eating Behavior Questionnaire-External Eating scale and BMI. The Tae-Eum type was found to have significantly higher SDFI and its subscale scores than the So-Eum type. Additionally, the SDFI-D subscale score was high in patients with diabetes mellitus and hypertension and low in functional dyspepsia and major depressive disease [20].

The sleep depicting psychophysical well-being [5] was also reported to show type-specific clinical characteristics in the Sasang typology [19]. The So-Eum type has issues with maintaining sleep owing to their high emotional instability [5, 21], the So-Yang type experiences difficulties in falling asleep, and the Tae-Eum type has frequent sleep apnea [16]. The perspiration governed by the sympathetic nervous system is known to be scarce in the Tae-Eum type. In addition, the sympathetic nervous system reactivity was suggested to be low in the Tae-Eum type and high in the So-Eum type [11, 19].

The Sasang Urination and Defecation Inventory (SUDI) [22] was developed to analyze Sasang type-specific urination and defecation excretory functions based on the results of systematic review [18]. Its subscales for investigating urination were found to have correlations with established clinical measures of the Urogenital Distress Inventory (UDI-6) and Overactive Bladder Symptom Score (OABSS), and significant subscale score differences between the Sasang types were suggested [22]. However, its use is still limited by the lack of clinical studies involving hospital patients and structural validation of the instrument.

The reliable and precise clinical measure of the pathophysiological symptoms is imperative for pattern identification of the Sasang type and Cold-Heat subgroup in the Sasang typology. The Sasang Urination Inventory (SUI) acquired from revising urinary function-related subscales of the SUDI might be an essential and practical assessment for the deterrence of exacerbations and confirmation of remissions in clinics [2, 11].

Therefore, the current study presented the Sasang type- and Cold-Heat subgroup-specific clinical features in urinary function after confirming the structural validity of the SUI using 3 years of records from hospital patients. The clinical differences in pathological urinary symptoms between the Sasang types and Cold-Heat subgroup diagnosed by a certified clinical specialist [23] were examined using the analysis of variance (ANOVA) and *T*-test after the SUI update using factor analysis and item response theory [24].

The objective clinical profiles of the urinary pathophysiological symptoms in the three Sasang types and their Cold-Heat subgroup might be acquired using well-validated clinical measures of the Sasang typology scales as previously shown with the SDFI, SPQ, BMI, and PI [3, 23, 25]. Furthermore, it would provide foundations for the evidence-based teaching [26] of the Sasang typology for medical students and professionals and for integrative precision medicine [6] combining Eastern and Western practices.

## 2. Methods and Materials

### 2.1. Participants and Procedures

Hospital records of 350 inpatients and outpatients of the Pusan National University Korean Medicine Hospital from October 2016 to August 2019 were included for the current analysis. The sex, age, height, weight, Sasang type, Cold-Heat subgroup, and responses to the SUDI questionnaire items acquired at the time of outpatient visit or hospitalization were retrieved and analyzed.

The current study was approved by the hospital institutional review board (PNUKHIRB-E2019008) as a retrospective study. Clinical data that could identify participants were carefully excluded, and the Declaration of Helsinki involving human subjects was followed.

### 2.2. Sasang Type and Cold-Heat Subgroup Classification of the Patients

The Sasang type and Cold-Heat subgroup identification of the patients were made by three certified clinical specialists of Sasang medicine (YGP, NB, and JYL) based on body shape, clinical responses to type- and subgroup-specific acupuncture treatment and herbal medication, and responses to the Sasang Personality Questionnaire for psychological characteristics, Sasang Digestive Function Inventory for Sasang type-specific pathophysiological features, and Questionnaire for Sasang Constitution Classification II for objective diagnosis of Sasang type. There were clear documentation showing improvement of the main complaints and no significant adverse events after type- and subgroup-specific medical decoctions for more than 28 days [7, 27].

### 2.3. Pathological Characteristics of Urinary Function

The pathological characteristics of participants' urinary function were measured using 13 items of the Sasang Urination and Defecation Inventory (SUDI) developed using a systematic review [18] and a clinical study [22]. The Sasang Urination Inventory (SUI, Table 1) in the current study is composed of 12 items selected from the SUDI for pathological characteristic analysis of urine in terms of color and odor (SUI-CHR or SUDI-CHR), hypersensitivity of increased frequency and urinal urgency (SUI-HSS) or reversed score of ability to hold urine (SUDI-RET), and discomforts in micturition (SUI-DIS or SUDI-DIS) using a 5-point Likert scale from “not at all” (0) to “very much” (4). The sum of the three subscales (SUI-Total) represents urinary function deterioration or pathologic symptoms in micturition with the range of 0–48 points.

A previous study [22] showed that the SUDI subscales are correlated with the Urogenital Distress Inventory (UDI-6) [28] assessing clinical distress in lower urinary tract symptoms during urination and the Overactive Bladder Symptom Score (OABSS) [29] examining daytime frequency, nocturia, urgency, and urgency incontinence. The SUDI-CHR was positively correlated with the BMI (*r* = 0.376, *p* < 0.05), the SUDI-RET negatively correlated with the UDI-6 (*r* = −0.331, *p* < 0.05) and OABSS (*r* = −0.510, *p* < 0.01), and the SUDI-DIS positively correlated with the UDI-6 (*r* = 0.378, *p* < 0.01) and OABSS (*r* = 0.302, *p* < 0.05).

### 2.4. Anthropometric Features of the Participants

The height (cm) and weight (kg) of participants were measured at the time of hospital visit to hospital for the calculation of the body mass index (BMI, kg/m^2^) and Ponderal Index (PI, kg/m^3^). The BMI is an anthropometric index for obesity measure and growth hormone, while the PI is a corporal index for lean body mass and thyroid hormone. The BMI and PI were found to be high in the Tae-Eum type and low in the So-Eum type [22, 30].

### 2.5. Statistical Analysis

The participants' demographic features were examined using descriptive statistics. The sexual differences in age, height, weight, BMI, and PI were attested using *T*-test. The differences among the Sasang types were examined using the *χ*^2^ test for sex distribution and the ANOVA and Bonferroni or Dunnett's T3 post hoc analysis for age, body weight, BMI, and PI. The differences between the Heat and Cold subgroups of each Sasang type were analyzed using the *χ*^2^ test for sex distribution and *T*-test for age.

The subscale item deletion or modification regarding the SUI subscales were made following the results of explorative factor analysis (EFA), and the structural validity of SUI subscales were demonstrated using the item response theory (IRT) and confirmatory factor analysis (CFA) [24, 31]. The EFA using principal axis extraction and Kaiser normalization with varimax rotation were used, and the factor structure was determined considering theoretical interpretation, conceptual coherence, scree plot, and eigenvalue and cumulative variance in the factor loading matrix. The items with factor loading less than 0.5, cross-loading in two factors, and factor loaded in unexpected factor were carefully chosen for the revision, and 12 items were finally selected for the SUI and its three subscales of SUI-CHR, SUI-HSS, and SUI-DIS.

The IRT was used to examine the infit as information-weighted measure and the outfit as outlier-sensitive fit of SUI items. The infit and outfit between 0.5 and 1.5 were considered acceptable. The Pearson's correlation was also used to examine item-total score correlation. The mean and standard deviation, skewness, and kurtosis of the SUI were calculated for the test-level statistics. Estimate of Guttman's L2 and coefficient alpha were used for the reliability analysis, and those close to 1.0 were considered acceptable. The Separation Index for persons was used as scale quality statistics to examine the sensitivity for distinguishing between high and low scores, and it is considered acceptable at 2.0 or higher.

The structural validity of the 12-item SUI was examined using the CFA. As for the model fit index, minimum chi-square (CMIN), root mean square error of approximation (RMSEA), root mean square residual (RMR), Comparative Fit Index (CFI), Tucker–Lewis Index (TLI), and Akaike information criterion (AIC) were used [32]. The structural equation model is considered acceptable when the CMIN is less than the alternative models, the CMIN/DF is between 1 and 3, the RMSEA is less than 0.08, the RMR is less than 0.5, the CFI is greater than 0.9, the TLI is greater than 0.95, and the AIC is less than the alternative models. The unstandardized factor loading, standardized factor loading, and critical ratio of CFA were also inspected for the structural validity of SUI. Cronbach's *α* was used to analyze the internal consistency of SUI and its subscales.

The SUI and subscale scores of each Sasang types and Cold-Heat subgroup were examined [22]. Pearson's correlation was used to examine the correlation among SUI and its subscales, age, BMI, and PI. The significant differences in SUI and its subscales among the three Sasang type groups were analyzed using analysis of covariance (ANCOVA) considering the age, and post hoc analysis was performed using the Bonferroni and Dunnett's T3 considering the result of Levene's test. The differences of SUI and its subscales between the Heat and Cold subgroups were examined using ANCOVA considering the sex for the So-Yang and Tae-Eum type groups, and *T*-test for the So-Eum type group.

The results are described as mean with standard deviation or frequency (%), and the statistical significance was determined at the level of *p* < 0.05, *p* < 0.01, and *p* < 0.001. IBM SPSS 23.0 (IBM, Armonk, NY) was used for the statistical analysis in this study, except for the confirmatory factor analysis with IBM SPSS AMOS 22.0 (IBM, Armonk, NY) and item response theory with jMetrik 4.1.1 (J. Patrick Meyer, Charlottesville, VA) [31].

## 3. Results

### 3.1. Demographic Features of the Participants

The participants in current study were 350 hospital patients (132 male and 218 female). There were no significant (*t* = −0.135, *p*=0.893) differences in age between male (46.36 ± 13.36) and female (46.60 ± 15.76) patients. The male patients (170.10 ± 6.40 and 70.40 ± 11.79, respectively) were significantly higher (*t* = 17.335, *p* < 0.001) and heavier (*t* = 8.222, *p* < 0.001) than the female patients (158.70 ± 5.69 and 59.72 ± 11.77) in height and weight. There were no significant differences in BMI (*t* = 1.274, *p*=0.176) between male (24.27 ± 3.43) and female patients (23.70 ± 4.43); however, the male patients (14.28 ± 2.03) had significantly lower (*t* = −2.345, *p* < 0.05) PI than the female patients (14.96 ± 2.91).

There were no significant differences (*χ*^2^ = 2.450, *p*=0.294) in sex among the three Sasang types (Table 2); nevertheless, there were significant differences in age (*F* = 13.486, *p* < 0.001), weight (*F* = 47.902, *p* < 0.001), height (*F* = 5.763, *p* < 0.01), BMI (*F* = 63.092, *p* < 0.001), and PI (*F* = 53.137, *p* < 0.001) among the three Sasang types. The age of the So-Yang (52.57 ± 14.99) Sasang type was significantly (*p* < 0.05) higher than those of the Tae-Eum (45.19 ± 14.59) and So-Eum (42.46 ± 15.60) Sasang types. Thus, the age was included as a covariance for the analysis of differences in SUI and its subscales between the Sasang type groups.

The weight of the Tae-Eum type (71.77 ± 11.47) was significantly (*p* < 0.05) heavier than those of the So-Yang (60.52 ± 10.16) and So-Eum (58.39 ± 12.36) types. The height of the So-Yang type (160.83 ± 7.53) was significantly (*p* < 0.05) lower than those of the Tae-Eum (164.22 ± 8.29) and So-Eum (163.69 ± 8.29) types. The BMI and PI of the Tae-Eum (26.63 ± 4.03 and 16.28 ± 2.83, respectively), So-Yang (23.33 ± 3.10 and 14.53 ± 2.02), and So-Eum (21.66 ± 3.30 and 13.24 ± 1.92) types were in decreasing order with significant differences (*p* < 0.05). These results show that the PI has the same clinical usefulness as the BMI for differentiating the physical characteristics of each Sasang type (Table 2).

The demographic features of the Cold-Heat subgroup in each Sasang type were examined. There were no significant differences between the Heat and Cold subgroups of the So-Yang (52.35 ± 15.30 and 52.93 ± 14.66, *t* = 0.091 (*p*=0.847)), Tae-Eum (54.33 ± 15.66 and 47.20 ± 15.07, *t* = 1.903 (*p*=0.059)), and So-Eum (44.44 ± 16.27 and 40.20 ± 14.26, *t* = 1.496 (*p*=0.137)) Sasang types in age.

The Cold and Heat subgroup distribution for the Sasang types was significantly (*χ*^2^ = 11.279, *p*=0.004) different as shown in Table 2. The sex ratio of the Cold (18 male and 23 female) and Heat (16 male and 50 female) subgroups was significantly (*χ*^2^ = 4.509, *p* < 0.05) different in the So-Yang type. The sex ratio of the Cold (42 male and 32 female) and Heat (9 male and 40 female) subgroups was significantly (*χ*^2^ = 17.900, *p* < 0.05) different in the Tae-Eum type. However, the sex ratio of the Cold (22 male and 42 female) and Heat (25 male and 31 female) subgroups was not significantly (*χ*^2^ = 0.292, *p*=0.589) different in the So-Eum type. Therefore, the sex was included as a covariate for the analysis of differences in SUI and its subscales between the Heat and Cold subgroups of the So-Yang and Tae-Eum types.

### 3.2. Formulation of SUI Subscales Using Explorative Factor Analysis

The SUI subscales (Table 1) were formulated based on the factor loading of urinary function-related SUDI items following the EFA. The SUI-CHR has the same questionnaire items of SUDI-CHR since factor loading of items was between 0.705 and 0.768. Deletion of one item with low factor loading and addition of one item from other factors along with the existing items of SUDI-RET with factor loading from 0.683 to 0.858 made the SUI-HSS, whose operational definition is hypersensitivity with increased frequency and urinal urgency. The SUI-DIS, which has similar operational definition of the SUDI-DIS, has four items with the factor loading from 0.70 to 0.853.

The final version of SUI has 12 items with score range of 0–48, and the SUI subscales of SUI-CHR, SUI-HSS, and SUI-DIS have 4 items each with score range of 0–16.

### 3.3. Construct Validity of SUI Using Item Response Theory and Confirmatory Factor Analysis

The use of IRT on SUI 12 items showed that the range of infit was 0.73–1.31 and the outfit was 0.71–1.30, which were both acceptable. The correlation coefficient between items and total score were between 0.50 and 0.75. The test level statistics showed 0.1202, −0.1888, and 16.62 ± 7.28 for skewness, kurtosis, and SUI-Total score, respectively. Guttman's L2 and coefficient alpha estimates were 0.8541 and 0.8461, respectively, for the SUI reliability. The Separation Index for persons was 2.4055, which was acceptable.

The CFA showed that the modified three-factor model (Table 3) of SUI has an acceptable model fit index (CMIN = 139.968 (*p* < 0.001), CMIN/DF = 2.916, RMSEA = 0.074, RMR = 0.053, CFI = 0.939, TLI = 0.917, AIC = 199.968). Moreover, the values of factor loading were practically meaningful since the standardized factor loading of the SUI items was 0.467–0.854 (Table 4). The coefficients of inter-correlation among extracted three factors of the SUI were examined, and there were recognizable correlations. The SUI-CHR was correlated with the SUI-HSS (*r* = 0.417, *p* < 0.05) and SUI-DIS (*r* = 0.443, *p* < 0.05), and the SUI-HSS was correlated with the SUI-DIS (*r* = 0.637, *p* < 0.05).

The internal consistency of SUI-total, SUI-CHR, SUI-HSS, and SUI-DIS using Cronbach's alpha was 0.846, 0.774, 0.703, and 0.844, respectively (Table 1).

### 3.4. Revised SUI Subscale Scores of the Sasang Type Groups and Cold-Heat Subgroup

The correlation coefficient between the revised SUI and its subscales, age, BMI, and PI, was examined (Table 5). The SUI-total score was significantly correlated with the SUI-CHR (*r* = 0.714, *p* < 0.01), SUI-HSS (*r* = 0.084, *p* < 0.01), SUI-DIS (*r* = 0.841, *p* < 0.01), age (0.346, *p* < 0.01), BMI (*r* = 0.190, *p* < 0.01), and PI (*r* = 0.161, *p* < 0.01). The correlation coefficients among the three SUI subscales were 0.330–0.555. The results showed that the age was correlated with the SUI-CHR (*r* = 0.106, *p* < 0.05), SUI-HSS (*r* = 0.369, *p* < 0.01), and SUI-DIS (*r* = 0.329, *p* < 0.01), which might suggest the increase of problematic urinary function following the age.

The significant differences of SUI and its subscale scores between the Sasang types were analyzed using ANCOVA considering age (Table 6). There were significant (*F* = 5.078, *p* < 0.007) differences in the SUI-CHR, and the post hoc analysis showed that the Tae-Eum type (6.15 ± 2.96) had significantly higher score than the So-Yang type (5.17 ± 2.84). Though the So-Eum type (5.28 ± 2.76) had lower SUI-CHR score than the Tae-Eum type, however, the difference between the So-Eum and Tae-Eum types was only marginal (*p*=0.079) for the significance.

The significant differences in the SUI and its subscale scores between the Heat and Cold subgroups of each Sasang types were analyzed (Table 7 and Supplementary Table 1). There was a significant difference in the SUI-HSS between the Heat and Cold subgroups of the Tae-Eum type. Furthermore, the Heat subgroup (7.15 ± 2.95) was significantly (*F* = 5.421, *p*=0.022) higher than the Cold subgroup (5.82 ± 2.68) in Tae-Eum type.

These results might show that the SUI-CHR and SUI-HSS subscales are clinically useful for the differential diagnosis of the Sasang type and Cold-Heat subgroup, especially in the Tae-Eum type.

## 4. Discussion

The current study provided the Sasang type- and Cold-Heat subgroup-specific clinical features for clinical type and pattern identification using well-validated SUI (Table 1) that might conciliate the medical theory of Jema Lee [2, 18], previous studies [22], and clinical practice in a traditional Korean medical hospital.

The SUI [22] in the current study would contribute to the establishment of object analysis on clinical profiling of the Sasang typology along with SDFI [10] for pathophysiological symptoms of the digestive function and the SPQ [6, 7] for biopsychological characteristics of the Yin-Yang and Sasang typology. The reliable precision medicine of the traditional East-Asian medicine [3, 4] using the Sasang typology might be achievable using the distinctive clinical differences between the So-Yang and Tae-Eum types in the current SUI study, Tae-Eum and So-Eum types in SDFI [10, 23], and So-Eum and So-Yang types in SPQ [5, 6, 23]. Furthermore, the pathological characteristics of the Cold and Heat subgroups were provided with SUI, which might be used clinically in combination with the pathophysiological characteristics revealed with SDFI and SPQ [23].

As for the physical characteristics, the PI presented clear differences in BMI between the Sasang types (Table 2) [30, 33], and the PI and BMI had high correlation coefficient (*r* = 0.956, *p* < 0.01) in the current study. The PI representing physical development and lean body mass might substitute BMI [22, 30] considering that the physical features of the Tae-Eum type were described as big and long, rather than obese and fat [2], compared with the So-Eum type in Jema Lee's original book, and the physical differences among the Sasang types disappeared when the BMI was considered as a covariate [33]. Further studies in consideration of the diverse demographic and sociocultural background would be needed to endorse the PI as a corporal index of Sasang typology.

The reliability and stability of SUI and its subscales were notable, and the IRT and factor analysis (Tables 3 and 4) were adopted for revising clinical measures of the Sasang typology for the first time.

First, the operational definition of SUI subscale was restructured for the clinical practicality [22], and the SUI-total score for measuring deteriorated urinary function was newly provided (Table 1). The SUDI-UCHR and SUDI-UDIS subscales maintained their label and definition as SUI-CHR and SUI-DIS subscales, while the SUDI-URET subscale was revised as SUI-HSS for measuring pathological hypersensitivity of urinal urgency and frequent micturition using reverse coding. Therefore, the SUI-DIS, SUI-HSS, and SUI-total scores of the current study would show positive correlation with the UDI-6 [28] of clinical distress in lower urinary tract symptoms during the urination and OABSS [29] of daytime frequency, nocturia, urgency, and urgency incontinence.

Second, the reliability and validity of SUI and its subscales were significantly improved. The internal consistency of current SUI-total, SUI-CHR, SUI-HSS, and SUI-DIS was 0.846, 0.774, 0.703, and 0.844, respectively, and those were markedly higher than the scores of 0.717, 0.554, and 0.625 for the SUDI-CHR, SUDI-RET, and SUDI-DIS, respectively [22]. The CFA showed the range of standardized factor loading for the SUI-CHR, SUI-HSS, and SUI-DIS as 0.495–0.789, 0.467–0.690, and 0.625–0.854, respectively, which were acceptable (Table 4). Moreover, the IRT revealed the range of infit and outfit to be 0.73–1.31 and 0.71–1.30, respectively, and the Separation Index for persons to be 2.4055, which demonstrate that the SUI and its items were proper for measuring the pathological characteristics and discriminating clinical differences of the participants. These statistical analyses are imperative for securing validation of clinical measure [24, 31, 34], and the current study is a prerequisite for providing reliable clinical measures of the traditional East-Asian medicine.

Along with the improved structural validity, the SUI and its subscales were also found to have clinical usefulness as follows. First, the SUI-total score was found to have positive correlations with the SUI-CHR (*r* = 0.714, *p* < 0.01), SUI-HSS (*r* = 0.804, *p* < 0.01), and SUI-DIS (*r* = 0.841, *p* < 0.01), and the range of correlation coefficient between SUI subscales was 0.33–0.56 (Table 5) compared with 0.235–0.334 of the SUDI. The operational definition of SUI and its subscales was well organized to measure the pathological problems in urinary function.

Interestingly, the age was positively correlated with the SUI-total (*r* = 0.346, *p* < 0.01), SUI-CHR (*r* = 0.106, *p* < 0.05), SUI-HSS (*r* = 0.369, *p* < 0.01), and SUI-DIS (*r* = 0.329, *p* < 0.01), which might show that the pathological issues with urinary function were increased following the age (Table 5).

Second, the SUI-CHR of physical characteristics of urine revealed distinctive differences between the So-Yang (5.17 ± 2.84) and Tae-Eum (6.15 ± 2.96) types (Table 6). Furthermore, the SUI-HSS and SUI-DIS subscale scores of the So-Yang (7.06 ± 3.14 and 4.80 ± 3.24, respectively) type were higher than the So-Eum (6.48 ± 3.27 and 4.13 ± 3.46) type as shown in the previous study using the small group of healthy participants [22]. However, there were no significant differences and demographic characteristics of the previous and current studies that might explain the discrepancies.

The clinical measure of the Sasang type-specific pathophysiological symptom discriminating the So-Yang type from the Tae-Eum type would complete the Sasang type identification system, since the SDFI [10, 23] presented distinctive clinical differences between the So-Eum and Tae-Eum types and the SPQ [5, 6, 23] showed significant clinical differences between the So-Eum and So-Yang types in previous clinical studies. The clinical profile [3, 24] of each Sasang type using the Sasang type-specific pathophysiological symptoms and biopsychological temperament might be completed with the current and further studies.

Third, the SUI-HSS was found to be useful in quantitatively presenting the clinical differences between the Cold-Heat subgroup. The Heat subgroup of the Tae-Eum type has significantly (*F* = 5.421) high SUI-HSS score showing hypersensitivity than the Cold subgroup (Table 7 and Supplementary Table 1), and this might be used clinically for pattern identification of the Cold-Heat subgroup in combination with previous findings [23] that the Heat subgroup has high score in SDFI-Digestion subscale (*F* = 4.123) of activated digestive function [10, 20], BMI (*F* = 21.907) of bodily obesity, and SPQ-Cognition subscale (*F* = 4.219) of proactive, and sociable and flexible cognitive style [7, 8].

The Cold-Heat pattern was originally suggested by the Yellow Emperor's Classic of Internal Medicine and Discussion on Cold-Induced Disease and has been the widely used pivotal clinical differentiation pattern of East-Asian traditional medicine until the ICD-11 [23, 35–37]. The Cold-Heat pattern of traditional East-Asian medicine refers to momentary and transient clinical manifestations of the patients including cold and fever, absence and presence of thirst, loose stool and constipation, and preference for heat and cold, [38] which might be the subjective perception of cold or heat [23, 35, 37].

However, the Cold-Heat subgroup of the Sasang typology denotes intrinsic and sustained biopsychological and pathophysiological predispositions, and previous studies showed that actual body temperature [39], BMI [23, 40, 41], and activated digestive function [39, 41, 42] and biopsychological activity [8, 23] might be the representative clinical measure. The BMI was suggested as the most important clinical feature for both Cold-Heat and Deficiency-Excess pattern identification by clinicians [35], and the pathological problem of urination (SUI) in the current study [22] was suggested to be related to the Pediatric Weak Scale-GN subscale representing Blood Heat pattern [24]. The Heat subgroup of the Sasang typology would be defined as a person with activated bodily and mental function [23] with the characteristics of Heat and Excess clinical patterns altogether, while the Cold subgroup has the inhibited or less activated biopsychosocial functioning with Cold and Deficiency patterns.

Although the current study provided notable clinical data, there still are limitations for the generalizability. First, the age of the 350 participants (Table 1) was relatively high at 46.51 ± 15.59, and further studies using diverse age groups including younger participants would be needed. Although the age and sex would not be influential factors since these are considered enough in the statistical analysis, the age and sex differences might have indirectly modulated the magnitude of differences between the Sasang types and Cold-Heat subgroup considering the finding that the age is significantly correlated with the SUI-total (*r* = 0.346, *p* < 0.01) and its subscales.

Second, there is a need for using other Sasang type classification methods including the Sasang Constitution Analysis Tool [43], Korea Sasang Constitutional Diagnostic Questionnaire [44], Two-Step Questionnaire for the Sasang Constitution Diagnosis [45], and Questionnaire Sasang Constitution Classification II [22] to confirm the findings in the current study. However, the differences in SUI among the Sasang types and Cold-Heat subgroup might be replicated as shown in the current study, since the diagnosis of the Sasang types was performed by the certified clinical specialists in this study.

Third, the correlation between SUI and well-validated measures of biomedicine including the UDI-6 and OABSS in clinical patients would be needed. Though the revision might not have caused significant changes of SUI subscales considering the similarity of subscales of SUI and SUDI, the expected positive correlation between the SUI and its subscales and western measures of the UDI-6 [28] and OABSS [29] should be confirmed. Moreover, the correlation among the subscales of SUI, SDFI [10, 46], SPQ [7], BMI, and PI [30] would be needed to establish a foundation for fabricating clinical profiles of each Sasang type and Cold-Heat subgroup.

Lastly, there were marginal (*p*=0.079) difference between the Tae-Eum (6.15 ± 2.96) and So-Eum (5.28 ± 2.76) types in the SUI-CHR along with the significant (*p* < 0.05) differences between the Tae-Eum (6.15 ± 2.96) and So-Yang (5.17 ± 2.84) types in the current study (Table 6). The demographic features of age and sex of participants might have prevented the difference between the Tae-Eum and So-Eum types statistically significant (Supplementary Table 1), and the uneven distribution (Table 7) of participants of Sasang type and Cold-Heat group might have hindered revealing notable differences. Further studies with bigger sample size would be required.

The current study identified distinctive clinical features of the Sasang types and Cold-Heat subgroup in urinary function using the well-designed and validated SUI. The foundation for precise diagnosis of the Sasang typology might be possible with the SUI used in current study, which may be useful for the evidence-based education and objective clinical practice of the Sasang typology.

## Figures and Tables

**Table 1:** Operational definition of the Sasang Urination Inventory subscales.

Subscale	Description and symptoms	No. of items	Cronbach's *α*
SUI-total	The sum of the CHR, HSS, and DIS subscales, which represent deterioration of urinary function	12	0.846
SUI-CHR	Problematic physical characteristics of urine. The urine has dark or yellowish color, high turbidity, strong odor, and foamy features.	4	0.774
SUI-HSS	Increased frequency and decreased volume in urination. There is frequent urination (day and night) and nocturia, decreased amount of urine at one time due to the frequent micturition, and frequent urinary urgency.	4	0.703
SUI-DIS	Urinary discomfort or discomfort micturition. There is a frequent experience of hesitancy, straining and residual urine sense (feeling of incomplete urination), and decreased total volume of urine for one day.	4	0.844

SUI, Sasang Urination Inventory; SUI-CHR, SUI-characteristics of urine; SUI-HSS, SUI-hypersensitivity of the urinary bladder; SUI-DIS, SUI-discomfort in micturition; BMI, body mass index; PI, Ponderal Index.

**Table 2:** Demographic characteristics of the participants in the current study.

	So-Yang	Tae-Eum	So-Eum	Total	Statistical analysis
Sex (m/f)	107 (34/73)	123 (51/72)	120 (47/73)	350 (132/218)	*χ* ^2^ = 2.450, *p*=0.294
Subgroup (Cold/Heat)	107 (41/66)	123 (74/49)	120 (64/56)	350 (179/171)	*χ* ^2^ = 11.279, *p*=0.004
Age^*∗∗∗*^	52.57 ± 14.99	45.19 ± 14.59	42.46 ± 15.60	46.51 ± 15.59	*F* = 13.486, *p* < 0.001, SY > TE & SE
Weight^*∗∗∗*^	60.52 ± 10.16	71.77 ± 11.47	58.39 ± 12.36	63.74 ± 12.85	*F* = 47.902, *p* < 0.001, TE > SY & SE
Height^*∗∗*^	160.83 ± 7.53	164.22 ± 8.29	163.69 ± 8.29	163.00 ± 8.14	*F* = 5.763, *p* < 0.01, TE & SE > SY
BMI^*∗∗∗*^	23.33 ± 3.10	26.63 ± 4.03	21.66 ± 3.30	23.92 ± 4.09	*F* = 63.092, *p* < 0.001, TE > SY > SE
PI^*∗∗∗*^	14.53 ± 2.02	16.28 ± 2.83	13.24 ± 1.92	14.70 ± 2.63	*F* = 53.137, *p* < 0.001, TE > SY > SE

SY, So‐Yang; TE, Tae‐Eum; SE, So‐Eum

**Table 3:** Model fit index for the revised SUI and its subscales.

Factor structure	CMIN	DF	CMIN/DF	RMSEA	RMR	CFI	TLI	AIC
3 factor	202.058	51	3.962	0.092	0.072	0.901	0.871	256.058
3 factor modified 1	170.241	50	3.962	0.083	0.063	0.921	0.895	226.241
3 factor modified 2	**139.968**	**48**	**2.916**	**0.074**	**0.053**	**0.939**	**0.917**	**199.968**

CMIN, minimum chi-square; DF, degree of freedom; RMSEA, root mean square error of approximation; RMR, root mean square residual; CFI, Comparative Fit Index; TLI, Tucker–Lewis Index; AIC, Akaike information criterion.

**Table 4:** Factor loading of the revised SUI items using confirmatory factor analysis.

Subscales	Unstandardized factor loading	Standard error	Critical ratio	Standardized factor loading
SUI-CHR				
Item 1	1.000	—	—	0.495
Item 2	1.509	0.176	8.596^*∗∗∗*^	0.672
Item 3	1.957	0.251	7.808^*∗∗∗*^	0.720
Item 4	1.726	0.218	7.903^*∗∗∗*^	0.789

SUI-HSS				
Item 9	1.000	—	—	0.467
Item 10	1.883	0.259	7.260^*∗∗∗*^	0.676
Item 11	1.827	0.250	7.309^*∗∗∗*^	0.690
Item 12	1.377	0.194	7.100^*∗∗∗*^	0.638

SUI-DIS				
Item 5	1.000	—	—	0.625
Item 6	1.461	0.119	12.271^*∗∗∗*^	0.854
Item 7	1.334	0.112	11.918^*∗∗∗*^	0.809
Item 8	1.464	0.130	11.28^*∗∗∗*^	0.746

^*∗∗∗*^ *p* < 0.001.

**Table 5:** Correlation coefficient between the revised SUI and its subscales, age, BMI, and PI.

	SUI-CHR	SUI-HSS	SUI-DIS	Age	BMI	PI
SUI-total	**0.714** ^*∗∗*^	**0.804** ^*∗∗*^	**0.841** ^*∗∗*^	**0.346** ^*∗∗*^	0.190^*∗∗*^	0.161^*∗∗*^
SUI-CHR		**0.330** ^*∗∗*^	**0.400** ^*∗∗*^	0.106^*∗*^	0.147^*∗∗*^	0.147^*∗∗*^
SUI-HSS			**0.555** ^*∗∗*^	**0.369** ^*∗∗*^	0.113^*∗*^	0.117^*∗*^
SUI-DIS				**0.329** ^*∗∗*^	0.130^*∗*^	0.118^*∗*^

Age					0.157^*∗∗*^	0.250^*∗∗*^
BMI						**0.956** ^*∗∗*^

Bold represents correlation coefficient over 0.3. ^*∗*^*p* < 0.05; ^*∗∗*^*p* < 0.01.

**Table 6:** The SUI and its subscale scores for each Sasang type group.

	So-Yang	Tae-Eum	So-Eum	Statistical analysis
SUI-total	17.03 ± 7.43	16.97 ± 6.82	15.89 ± 7.63	*F* = 0.818, *p*=0.442
SUI-CHR^*∗*^	5.17 ± 2.84	6.15 ± 2.96	5.28 ± 2.76	*F* = 5.078, *p* < 0.007, So-Yang < Tae-Eum
SUI-HSS	7.06 ± 3.14	6.62 ± 2.91	6.48 ± 3.27	*F* = 0.107, *p*=0.899
SUI-DIS	4.80 ± 3.24	4.20 ± 3.03	4.13 ± 3.46	*F* = 0.057, *p*=0.945

**Table 7:** The SUI and its subscale scores for the Cold and Heat subgroups in each Sasang type.

	Cold subgroup	Heat subgroup	Statistical analysis
So-Yang (male/female)	(18/23)	(16/50)	
SUI-total	17.08 ± 7.26	16.95 ± 7.79	*F* = 0.118, *p*=0.732
SUI-CHR	5.11 ± 2.93	5.27 ± 2.73	*F* = 0.020, *p*=0.888
SUI-HSS	7.21 ± 2.99	6.80 ± 3.40	*F* = 0.276, *p*=0.601
SUI-DIS	4.76 ± 3.22	4.88 ± 3.32	*F* = 0.024, *p*=0.878

Tae-Eum (male/female)	(42/32)	(9/40)	
SUI-total	15.20 ± 6.62	18.14 ± 6.75	*F* = 1.482, *p*=0.226
SUI-CHR	5.63 ± 2.56	6.50 ± 3.16	*F* = 0.074, *p*=0.786
SUI-HSS^*∗*^	5.82 ± 2.68	7.15 ± 2.95	*F* = 5.421, *p* < 0.022
SUI-DIS	3.76 ± 3.05	4.49 ± 3.00	*F* = 0.453, *p*=0.502

So-Eum (male/female)	(22/42)	(25/31)	
SUI-total	15.81 ± 7.76	15.98 ± 7.56	*t* = −0.121, *p*=0.904
SUI-CHR	5.30 ± 2.80	5.25 ± 2.74	*t* = 0.092, *p*=0.926
SUI-HSS	6.50 ± 3.21	6.46 ± 3.37	*t* = −0.397, *p*=0.953
SUI-DIS	4.02 ± 3.46	4.27 ± 3.49	*t* = −0.121, *p*=0.692

## Data Availability

The data are available from the corresponding author upon request.

## Supplementary Materials

Supplementary Materials Supplementary Table 1. Age, sex and BMI adjusted mean and standard error of SUI and its subscales.
