# Supplementary material for: Urinary Function of the Sasang Type and Cold-Heat Subgroup Using the Sasang Urination Inventory in Korean Hospital Patients
Source: Evid Based Complement Alternat Med. 2020 Sep 9;2020:7313581. doi: 10.1155/2020/7313581 (PMC7499285; doi:10.1155/2020/7313581)
Supplement: Supplementary Materials — Supplementary Table 1. Age, sex and BMI adjusted mean and standard error of SUI and its subscales. [file 7313581.f1.docx]

**Supplementary Table 1.** Age, sex and BMI adjusted mean and standard error of SUI and its subscales.

|  | Cold subgroup | Heat subgroup |
| --- | --- | --- |
| So-Yang (male/female) | (18/23) | (16/50) |
| SUI-total | 17.27±0.91 | 16.63±1.16 |
| SUI-CHR | 5.21±0.35 | 5.1±0.45 |
| SUI-HSS | 7.2±0.37 | 6.82±0.48 |
| SUI-DIS | 4.86±0.39 | 4.71±0.5 |
| Tae-Eum (male/female) | (42/32) | (9/40) |
| SUI-total | 16.65±0.84 | 17.18±0.67 |
| SUI-CHR | 6.33±0.39 | 6.04±0.31 |
| SUI-HSS* | 6.06±0.39 | 6.99±0.31 |
| SUI-DIS | 4.27±0.4 | 4.15±0.32 |
| So-Eum (male/female) | (22/42) | (25/31) |
| SUI-total | 15.62±0.91 | 16.2±0.97 |
| SUI-CHR | 5.35±0.32 | 5.19±0.35 |
| SUI-HSS | 6.36±0.4 | 6.62±0.43 |
| SUI-DIS | 3.92±0.42 | 4.38±0.45 |
